# A Mistake to Exercise for Strength Alone

**Published:** 1890-03

**Authors:** 


### A MISTAKE TO EXERCISE FOR STRENGTH ALONE.

When great muscular strength or agility follows in the wake of physical exercise, these should be regarded as incidental and entirely subordinate to the health of body which the exercise has secured. To exercise for strength alone, and to estimate it as the chief aim is an inexcusable blunder. There is no necessary physiological, casual relation between strength and health. Indeed it is a notorious fact that professional athletes are often defective in some bodily organ, and they generally die early in life from either heart or lung trouble. Developing certain sets of muscles to the exclusian of others makes the muscular system unsymmetrical, and interferes with the equable distribution of the general blood supply. Inordinate development of muscular power calls for unnatural activity from the central vital organs, and thus it frequently occurs that under the strain of some special effort the heart or lungs fail, and death results.—*The Doctor.*

